# Design and Synthesis of Vandetanib Derivatives Containing Nitroimidazole Groups as Tyrosine Kinase Inhibitors in Normoxia and Hypoxia

**DOI:** 10.3390/molecules21121693

**Published:** 2016-12-14

**Authors:** Huiqiang Wei, Deguan Li, Xiangbo Yang, Haihua Shang, Saijun Fan, Yiliang Li, Dan Song

**Affiliations:** 1 Tianjin Key Laboratory of Radiation Medicine and Molecular Nuclear Medicine, Institute of Radiation Medicine, Peking Union Medical College & Chinese Academy of Medical Sciences, Tianjin 300192, China; wakie0208@163.com (H.W.); ldguan@163.com (D.L.); shanghaihua2005@163.com (H.S.); fansaijun@163.com (S.F.); 2 Chongqing Technical Center for Drug Evaluation & Certification, Chongqing 400014, China; 3 School of Petrochemical Engineering, Changzhou University, Changzhou 213164, China; 15122363480@163.com

**Keywords:** tyrosine kinase inhibitor, vandetanib, nitroimidazole, hypoxia

## Abstract

Sixteen novel epidermal growth factor receptor (EGFR)/vascular endothelial growth factor (VEGF)-2 inhibitors (nitroimidazole-substituted 4-anilinoquinazoline derivatives (**16a**–**p**)) were designed and prepared via the introduction of a nitroimidazole group in the piperidine side chain and modification on the aniline moiety of vandetanib. Preliminary biological tests showed that comparing with vandetanib, some target compounds exhibited excellent EGFR inhibitory activities and anti-proliferative over A549/H446 cells in hypoxia. Meanwhile, several of the above compounds demonstrated better bioactivity than vandetanib in VEGF gene expression inhibition. Owing to the excellent IC_50_ value (1.64 μmol/L), the inhibition ratios of **16f** over A549 and H446 cells were 62.01% and 59.86% at the concentration of 0.5 μM in hypoxia, respectively. All of these results indicated that **16f** was a potential cancer therapeutic agent in hypoxia and was worthy of further development.

## 1. Introduction

Hypoxia is a common phenomenon in most solid tumors [1,2]. Hypoxic tumor cells are distant from blood vessels and general drugs are difficult to enter internal hypoxic tissues [2]. Hypoxic tumor cells are resistant to radiotherapy and liable to transform from the G0 phase into normal proliferative ones after oxygen concentration increased [3]. Moreover, the expression of hypoxia-inducible factor-1 (HIF-1) can be promoted by hypoxia and enhance gene expression levels of some cell growth factors [4,5,6] and tyrosine kinase receptors, such as vascular endothelial growth factor (VEGF), platelet-derived growth factor-β (PDGF-β), and epidermal growth factor receptor (EGFR) [7]. These cell growth factors and tyrosine kinase receptors induce a series of cellular circumstances, such as angiogenesis, tumor cells proliferation and migration, through tyrosine kinase signaling pathways [5]. Hence, traditional radiotherapy and chemotherapy are invalid towards hypoxic tumor cells. In recent years, multi-targeted TKIs and irreversible TKIs have been hot products on market, which are not effective towards hypoxic tumor cells, yet. Therefore, overcoming hypoxic resistance of solid tumors has become a tremendous challenge in cancer therapy [8].

Nitroimidazole derivatives have been developed as hypoxia radiosensitizers in radiotherapy [9,10] because of their high affinities to hypoxic tissues, specifically. As electron-affinic compounds, radiosensitizers fix the radiation-induced damages in DNAs and enhance the effects of radiotherapy [11,12]. Cytotoxic metabolites of nitroimidazole radiosensitizers in hypoxia are hydroxylamines, nitroso compounds, and amines, which are liable to bind to DNAs irreversibly and lethal to tumor cells [13]. Now nitroimidazole derivatives have been used as hypoxia radiotracers labeled with ^99m^Tc, ^68^Ga and ^18^F for tumor hypoxic regions imaging [14,15,16,17]. The DNA alkylating agent Evofosfamine (TH-302) also contains a 2-nitroimidazole group and targets to hypoxic tissues in soft tissue sarcoma (STS) and pancreatic adenocarcinoma [18]. Based on the above studies, we conceived novel 4-anilinoquinazoline tyrosine kinase inhibitors containing nitroimidazole and anticipated their efficient antitumor activities both in normoxia and hypoxia.

**Figure 1:**
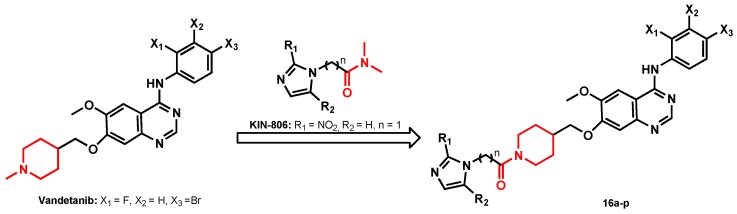
Structures of vandetanib, KIN-806, and target compounds.

In this study, vandetanib (Figure 1) was chosen as a small molecule tyrosine kinase inhibitor targeted to EGFR/VEFR-2, and 2-nitroimidazole hypoxia radiosensitizer KIN-806 [19] (Figure 1) as lead compounds. According to previous researches [20], substituted 4-anilinoquinazoline scaffold in vandetanib is the crucial pharmacophore for EGFR/VEFR-2 inhibition, which donates key H-bonds by N1 atom of quinazoline moiety and inserts into the hydrophobic pocket by aniline moiety in corresponding crystal structures of EGFR and VEFR-2. The *N*-methyl-piperidine side chain of vandetanib is the kinetophore of compatibilities and we merged the amid group of KIN-806 and its analogues onto the piperidine N4 position in the side chain of vandetanib to obtain target compounds (Figure 1). Based on this strategy, sixteen target compounds were constructed and evaluated their molecular docking study as well as in vitro biological activities.

## 2. Results and Discussion

### 2.1. Chemistry

**Scheme 1:**
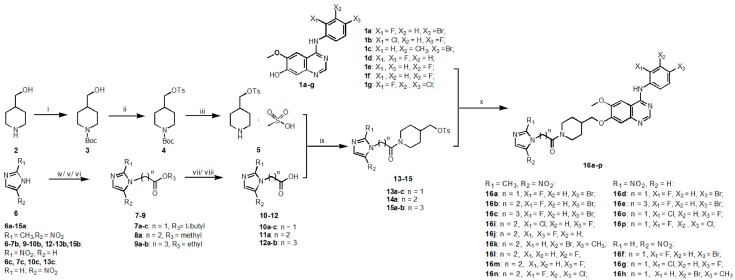
Synthesis of target compounds. *Reagents and conditions*: (**i**) (Boc)_2_O, MeOH, rt; (**ii**) *p*-TsCl, pyridine, 0 °C to rt; (**iii**) methylsulphonic acid, toluene, *i*-PrOH, 50 °C ; (**iv**) *t*-Butyl bromoacetate, K_2_CO_3_, ACN, reflux, (**7a**–**c**); (**v**) methyl acrylate, DIPEA, ACN, r.t., 7 days, (**8a**); (**vi**) Ethyl 4-bromobutyrate, K_2_CO_3_, ACN, reflux, (**9a**–**b**); (**vii**) TFA, CH_2_Cl_2_, r.t., (**10a**–**c**); (**viii**) OH^−^, 85 °C, 0.5–3 h, then H^+^, r.t., (**11a**, **12a**–**b**); (**ix**) CDI, TEA, DMF, r.t.; and (**x**) K_2_CO_3_, DMF, 85 °C.

Target compounds were synthesized from substituted 4-anilino-6-methoxyquinazoline (**1a**–**g**). The synthetic route displayed in Scheme 1 was started from 4-piperidine methanol (**2**) and nitroimidazoles (**6a**–**6c**). The *p*-toluene sulfonyl substituted 4-piperidinyl methanol mesylate (**5**) was prepared from **2** via Boc-protection, sulfonylation [21], and deprotection [22], as reported in early studies. Intermediates **7**–**9** (excepted for **8a**) were obtained with good yields (up to 96%) by reaction of nitroimidazoles (**6a**–**c**) with α-/β-/γ-bromo-carboxylic esters in the presence of base (K_2_CO_3_) in refluxing acetonitrile; **8a** was prepared from methyl acrylate and **6a** by Michael addition with *N*,*N*-diisopropylethylamine (DIPEA) as base in acetonitrile at room temperature with a good yield of 95.6%. The 2-(substituted-nitroimidazol-1-yl)acetic acid (**10a**–**c**) were prepared by acidic hydrolysis in 30% TFA/CH_2_Cl_2_ solution; 3-(substituted-nitroimidazol-1-yl)propionic acid (**11a**) and 4-(substituted-nitroimidazol-1-yl)butyric acid (**12a**–**b**) were prepared by alkaline hydrolysis in saturated Na_2_CO_3_ solution at 85 °C for 2.5 h and then acidizing with 6 N HCl solution. Catalyzed by carbonyldiimidazole (CDI) and triethylamine (TEA), the key intermediates **13**–**15** were synthesized from **5** and **10**–**12** in DMF at room temperature with acceptable yields varying from 78%–91%. Target compounds **16a**–**p** were obtained by intermediates **13**–**15** connecting with **1a**–**g** in the presence of K_2_CO_3_ in DMF at 85 °C with moderate yields of 52%–76%.

### 2.2. Molecular Docking Study

**Figure 2:**
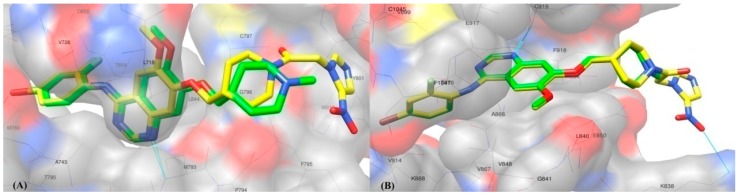
The docked poses of compound **16f** (C: yellow; N: blue; O: red) or vandetanib (C: green; N: blue; O: red) at the ATP binding cleft of EGFR kinase (**A**), and VEGFR-2 kinase (**B**).

To predict the possible binding mode of target compounds with EGFR and VEFR-2, two docking studies of compound **16f** were performed using vandetanib as a comparison. As illustrated in Figure 2, the crystal structure of the complex between **16f** and EGFR (Figure 2A) reveals that N1 atom of quinazoline moiety formed a H-bond with the backbone-NH of Met793 with a distance of 2.823 Å in a similar way to vandetanib, and the aniline moiety inserted into the hydrophobic pocket but did not form any additional H-bonds; In another crystal structure complex between **16f** and VEFR-2 (Figure 2B), **16f** favorably fitted into VEFR-2 with the aniline moiety occupying the narrow deep hydrophobic pocket and a critical H-bond between quinazoline N1 atom and Cys919 with a distance of 3.099 Å and it was in a similar way to vandetanib. As we expected, the nitroimidazole side chain was positioned at the solvent portion of the kinase. However, an extra H-bind was located at the opening of the hydrophobic pocket in the crystal structure complex between **16f** and VEFR-2, which formed between the nitro group and the side chain amino group of Lys838 residue with an non-normal distance of 3.491 Å. We inferred that halogen atoms on the aniline moiety contributed more hydrophobicity and electronegativity, which caused a reinforced affinity to corresponding receptors; from another aspect, since the nitroimidazole side chain was positioned at the solvent portion of the kinase, advisable bigger and heavier halogen atoms on aniline would balance the “barycenter” of target compounds and fixed it in the complex. Bioassay results have proved this.

### 2.3. Biological Evaluation

In vitro EGFR inhibitory activity, in vitro anti-proliferative activity in normoxia/hypoxia, and VEGF/EGF gene expression inhibitory activity of target compounds have been determined in this section. Human epidermoid carcinoma A431 cells and human non-small cell lung cancer H1975 cells were used in in vitro EGFR inhibitory activity assays of target compounds by standard (3-(4,5-dimethylthiazol-2-yl)-5-(3-carboxymethoxyphenyl)-2-(4-sulfophenyl)-2*H*-tetrazolium (MTS)) method with vandetanib as a comparison [23]. Human non-small cell lung cancer A549 cells, human lung cancer H446 cells and human cervical cancer Hela cells were used in in vitro normoxic/hypoxic anti-proliferative activity tests by CCK-8 method with admixing cobalt chloride to establish a hypoxic model. VEGF/EGF gene expression inhibitory assays in hypoxia and normoxia were referred to a previous literature [24] by quantitative polymerase chain reaction (Q-PCR).

#### 2.3.1. In Vitro EGFR Inhibitory Activity

Two EGFR over-expressed cell lines of human epidermoid carcinoma A431 cells and human non-small cell lung cancer H1975 cells (T790M mutation [25]) were used to evaluate the in vitro biological activities of EGFR inhibition by MTS method. As shown in Table 1, more than a half of target compounds demonstrated acceptable anti-proliferative activities towards A431 cells and **16f** showed the best IC_50_ value of 1.64 μmol/L, but all were inferior to vandetanib (IC_50_ = 0.85 μmol/L). However, **16a**–**p** exhibited undesirable inhibitory activities over T790M-mutated H1975 cells with IC_50_ values all over 40 μmol/L, showing much lower activities than vandetanib (IC_50_ = 4.81 μmol/L). 4-Bromo-2-fluoro and 3,4-dichloro-2-fluoro substituted compounds (**16a**–**f**, **16n** and **16p**) showed relatively better inhibitory activities than other substituted 4-anilinoquinazoline compounds; smaller and lighter fluorine atoms substituted compounds (**16j**, **16l**, and **16m**) all showed obvious lower inhibitory activities. The bioassay results coincided well with the aforementioned inference in a DOCK study. With higher single electron reduction potentials, 2-nitroimidazole derivatives showed more potent hypoxia radiosensitizing activity than 5(4)-nitroimidazole derivatives as radiosensitizers; side chain length would contribute to molecular flexibility efficiently, which might cause reduced activities and unanticipated toxicities. However, the results demonstrated that nitro group’s position and side chain length (no more than three carbon atoms) did not impact the inhibitory activity observably. It indicated that introducing different nitroimidazole substituents and different lengths of side chains to piperidine moiety of vandetanib did not alter its wild-type EGFR inhibitory activity drastically, but led to detrimental binding interactions with T790M mutant EGFR kinase. Nevertheless, the in vitro anti-tumor assays proved that target compounds remain normal EGFR inhibitory activities and nitroimidazole side-chains did not temper with the original EGFR-selective inhibitory activity.

#### 2.3.2. In Vitro Anti-Proliferative Activity Assays in Normoxia and Hypoxia

To confirm whether target compounds showed better anti-tumor activities in hypoxia than in normoxia, compounds **16a**–**f**, **16h**, **16i**, **16n**, and **16p** were tested the inhibition ratios at two concentrations (5 μmol/L and 0.5 μmol/L) over three cell lines (A549, H446 and Hela) through CCK-8 method in normoxia and hypoxia. Hypoxia in these assays was induced by 200 μmol/L cobalt chloride solutions. As shown in Table 2, A549 cells inhibition ratios of target compounds were close to H446 cells (except for **16d**) in identical conditions. In hypoxic conditions, nearly all compounds (except for **16d**) exhibited inhibition ratios approaching or exceeding 50%. **16b**, **16c**, and **16f** exhibited favorable anti-proliferative activities over both A549 and H446 cells at the concentration of 0.5 μmol/L. All target compounds demonstrated more potent inhibitory activities than vandetanib in hypoxic conditions at both concentrations, especially over A549 cells at the concentration of 0.5 μmol/L (except for **16d**). Inhibition ratios of target compounds in hypoxia were all significantly improved compared with normoxia (except for **16d** over A549 cells at 0.5 μmol/L), especially **16b** and **16f**. It was also observed in vandetanib groups, but not as obvious as mentioned above. The results demonstrated that the nitro group’s position did not impact the inhibitory activity observably in hypoxia. We deduced that the more potent inhibitory activities of target compounds in hypoxia were attributed to the cytotoxicities by the reductive activation of nitroimidazole groups, which could counteract the hypoxia-activated tumor cells to some extent. A recent research [26] has also proved it. However, all target compounds showed low inhibition ratios over HeLa cells at both two concentrations in normoxia and hypoxia, and only **16c** exhibited the best inhibition ratio barely more than 20% at low concentration in hypoxia. Compared with vandetanib, target compounds over HeLa cells (except for **16p**) showed evidently inferior inhibition ratios at high concentration and comparable inhibition ratios at low concentration, notwithstanding that none of them exhibited inhibitory activities over 40%.

#### 2.3.3. In Vitro VEGF/EGF Gene Expression Inhibitory Activity

We deemed that the cytotoxicities in nitroimidazole reductive activation were not the only reasons for the enhanced anti-tumor activities in hypoxia and it could not explain the low inhibition ratios over HeLa cells. Some cell growth factors such as VEGF and EGF played critical roles in tumor proliferation, survival, invasion, and migration [4]. We conceived that target compounds could impact their genes expression and promote tumor cell death. To validate the assumptions, we conducted a “black-box” experiment by testing in vitro VEGF/EGF gene expression levels in hypoxia and normoxia.

Hypoxia in these tests was induced by cobalt chloride and quantitative polymerase chain reaction (Q-PCR) was used to evaluate the expression levels of VEGF and EGF genes over A549 cells by testing VEGF and EGF mRNA amounts. In this assay, compounds **16a**–**f** were tested at the concentration of 5 μmol/L. As shown in Table 3, The VEGF mRNA levels of target compounds groups in hypoxia were all lower than control group and vandetanib group significantly by over 1000-fold and 400-fold, respectively. This indicated that all of the target compounds exhibited excellent VEGF gene expression inhibitory activities and were distinctly superior to vandetanib. As a comparison, VEGF gene expression levels of target compounds groups in normoxia were all higher than that in hypoxia by over 100-fold; VEGF gene expression level in normoxia were also higher that in hypoxia by nearly three-fold in the vandetanib group. However, EGF gene expression level assays exhibited nearly opposite results. In hypoxia, EGF gene expression levels raised to different extents (except for **16f**) and the results in vandetanib groups were augmented more obviously than that in target compounds groups. In normoxia, EGF gene expression level all decreased excepted for **16b** and **16e** showed a slightly increased expression level. In these assays, we confirmed that **16f** demonstrated all evident decreased expression levels in hypoxia and normoxia and showed the best inhibitory activities in both VEGF and EGF gene expression assays.

Hypoxia-inducible factor-1α (HIF-1α) became stabilized in hypoxic tumor tissues and combined with HIF-1β subunit to form the heterodimer of HIF-1, which could bind with and activate VEGF promoter as a transcription factor in the cell nucleus, enhancing the expression level of VEGF mRNA and inducing angiogenesis and metastatic processes in tumors [4]. However, the VEGF gene expression assays exhibited very distinct decreased expression levels in hypoxia, which were incompatible with what we predicted above. Moreover, the EGF gene showed different expression levels in hypoxia and normoxia. We inferred that target compounds impacted VEGF/EGF gene expression levels through influencing the signaling pathways by some unknown mechanisms and more correlational research is required in our further work.

## 3. Experimental Section

### 3.1. Materials and Methods

#### 3.1.1. General Methods

Reactions were monitored by thin-layer chromatography (TLC) using silica gel 60 UV254 pre-coated silica gel plates. Detection was by means of a UV lamp. Flash column chromatography was performed on 300–400 mesh silica gel. Melting points were determined on an X-5 melting-point apparatus (Taike technology Co., Ltd., Beijing, China) and were not corrected. ^1^H-NMR, ^13^C-NMR, and ^19^F-NMR were recorded on a BRUKER AVANCE III spectrometer (Bruker Ltd., Faellanden, Switzerland) at 600 MHz/151 MHz/564 MHz or an AV400 spectrometer at 400 MHz/100 MHz/376 MHz. Chemical shifts were reported in ppm using DMSO-*d*_6_ solution without TMS or CFCl_3_ as internal standards. Mass spectra (ESI-MS) were performed on Agilent 1200 HPLC-6310 liquid chromatography mass spectrometer (Agilent Ltd., Palo Alto, CA, USA). All reagents and solvents were used as received from commercial sources without further purification.

#### 3.1.2. Synthesis

##### *General Synthetic Procedure of tert-Butyl 2-(Substituted-nitroimidazol-1-yl)acetate* (**7a**–**7c**)

To a solution of **6a**–**c** (1 eq) in ACN was added K_2_CO_3_ (1.1 eq) and stirred for 0.5 h at 50 °C. Then a solution of tert-Butyl bromoacetate (1.2 eq) in ACN was added dropwise to the first solution. The resulting mixture was refluxed until no **6a**–**c** appeared on TLC. The reaction mixture was cooled to room temperature and filtered. The filtrate was evaporated under reduced pressure, and the residue was dissolved in a mixture of EA and water. The organic layer was separated and the aqueous layer was extracted twice. The organic extracts were combined, dried (anhydrous Na_2_SO_4_) and evaporated to dryness to afford crude **7a**–**7c** without further purification.

*tert-Butyl 2-(2-Methyl-5-nitroimidazol-1-yl)acetate* (**7a**). Following the general procedure. Yield: 95.6%. Faint yellow solid. m.p. 109.6–111.2 °C; ESI-MS *m*/*z* = 242.17 [M + H]^+^; ^1^H-NMR (400 MHz, DMSO-*d*_6_) δ 1.43 (s, 9H), 2.27 (s, 3H), 4.94 (s, 2H), 8.25 (s, 1H).

*tert-Butyl 2-(2-Nitroimidazol-1-yl)acetate* (**7b**). Following the general procedure. Yield: 94.6%. Faint yellow solid. m.p. 89.9–91.7 °C; ^1^H-NMR (400 MHz, DMSO-*d*_6_) δ 1.41 (s, 9H), 5.12 (s, 2H), 7.21 (s, 1H), 7.62 (s, 1H). Yield: 94.0%. White solid. m.p. 137.7–138.2 °C; ESI-MS *m*/*z* = 228.13 [M + H]^+^; ^1^H-NMR (400 MHz, DMSO-*d*_6_) δ 1.44 (s, 9H), 4.97 (s, 2H), 7.81 (s, 1H), 8.34 (s, 1H).

*tert-Butyl 2-(5-Nitroimidazol-1-yl)acetate* (**7c**). Following the general procedure. Yield: 94.0%; White solid. m.p. 137.7–138.2 °C; ESI-MS *m*/*z* = 228.13 [M + H]^+^; ^1^H-NMR (400 MHz, DMSO-*d*_6_): δ (ppm): 1.44 (s, 9H), 4.97 (s, 2H), 7.81 (s, 1H), 8.34 (s, 1H).

##### *Methyl 3-(2-Methyl-5-nitroimidazol-1-yl)propionate* (**8a**)

To a solution of **6a** (20g, 157.40 mmol, 1.0 eq) in DMF (100 mL) were added methyl acrylate (14.90 g, 173.14 mmol, 1.1 eq) and DIPEA (20.0 g, 157.40 mmol, 1.0 eq) under nitrogen atmosphere. The mixture was stirred at room temperature for **7d**. Then reaction mixture was evaporated under reduced pressure, and the residue was dissolved in mixture of EA (400 mL) and water (400 mL). The organic layer was washed by brine (200 mL × 2), dried (anhydrous Na_2_SO_4_) and evaporated to dryness to afford the product **8a** as brown oil (32.08 g, 95.6%). ESI-MS *m*/*z* = 214.09 [M + H]^+^; ^1^H-NMR (400 MHz, DMSO-*d*_6_) δ 2.37 (s, 3H), 2.92 (t, *J* = 6.8 Hz, 2H), 3.60 (s, 3H), 4.21 (t, *J* = 6.8 Hz, 2H), 8.29 (s, 1H).

##### *General Synthetic Procedure of Ethyl 4-(Substituted-nitroimidazol-1-yl)butyrate* (**9a**–**b**)

**9a**–**b** was prepared according to the synthetic procedure of **7a**–**c** from **6a**–**b.**

*Ethyl 4-(2-Methyl-5-nitroimidazol-1-yl)butyrate* (**9a**). Following the general procedure. Yield: 93.2%. Brown oil without further purification.

*Ethyl 4-(2-Nitroimidazol-1-yl)butyrate* (**9b**). Following the general procedure. Yield: 85.7%. Yellow solid. m.p. 69.3–70.9 °C; ESI-MS *m*/*z* = 227.11 [M + H]^+^; ^1^H-NMR (400 MHz, DMSO-*d*_6_) δ 1.15 (t, *J* = 7.2 Hz, 3H), 2.04 (m, 2H), 2.34 (t, *J* = 7.2 Hz, 2H), 4.00 (q, *J* = 7.2 Hz, 2H), 4.40 (t, *J* = 7.2 Hz, 2H), 7.16 (s, 1H), 7.64 (s, 1H).

##### *General Synthetic Procedure of 2-(Substituted-nitroimidazol-1-yl)acetic acid* (**10a**–**c**)

To a solution of CF_3_COOH (1 eq) in CH_2_Cl_2_ was added **7a**–**c** (1 eq). The mixture was stirred at room temperature for 2.5 h, and evaporated under reduced pressure. The residue was dissolved in the mixture solvent (EA/PE, 1:1, *v*/*v*) under vigorous stirring. The mixture was filtrated to afford the solid product **10a**–**c**.

*2-(2-Methyl-5-nitroimidazol-1-yl)acetic acid* (**10a**). Following the general procedure. Yield: 84.3%. White solid. m.p. 220.9–221.9 °C; ESI-MS *m*/*z* = 184.04 [M − H]^−^; ^1^H-NMR (400 MHz, DMSO-*d*_6_) δ 2.28 (s, 3H), 4.93 (s, 2H), 8.25 (s, 1H), 13.44 (br s, 1H).

*2-(2-Nitroimidazol-1-yl)acetic acid* (**10b**). Following the general procedure. Yield: 87.5%. Faint yellow solid. m.p. ~138 °C (decomposition); ESI-MS *m*/*z* = 170.03 [M − H]^−^; ^1^H-NMR (400 MHz, DMSO-*d*_6_): δ (ppm): 5.21 (s, 2H), 7.20 (s, 1H), 7.62 (s, 1H), 13.42 (br s, 1H).

*2-(5-Nitroimidazol-1-yl)acetic acid* (**10c**). Following the general procedure. Yield: 90.5%. White solid. m.p. 179.3–180.6 °C; ESI-MS *m*/*z* = 170.03 [M − H]^−^; ^1^H-NMR (400 MHz, DMSO-*d*_6_) δ 4.97 (s, 2H), 7.81 (s, 1H), 8.34 (s, 1H), 13.41 (br s, 1H).

##### *General Synthetic Procedure of 3-(Substituted-nitroimidazol-1-yl)propionic acid* (**11a**) *and 4-(Substituted-nitroimidazol-1-yl)butyric acid* (**12a**–**b**)

To a solution of saturated Na_2_CO_3_ solution was added **8a** (**9a**–**b**). The mixture was attired at 85 °C for 2.5 h and then cooled to room temperature. The pH of the reaction solution was adjusted to 4, and the solid precipitated out. The crude product (**11a**, **12a**–**b**) was obtained by filtration, washed with EA and dried in the vacuum.

*3-(2-Methyl-5-nitroimidazol-1-yl)propionic acid* (**11a**). Following the general procedure. Yield: 87.5%. White solid. m.p. 204.9–206.3 °C; ESI-MS *m*/*z* = 198.06 [M − H]^−^; ^1^H-NMR (400 MHz, DMSO-*d*_6_) δ 2.37 (s, 3H), 2.81 (t, *J* = 6.8 Hz, 2H), 4.16 (t, *J* = 6.8 Hz, 2H), 8.28 (s, 1H), 12.54 (br s, 1H).

*4-(2-Methyl-5-nitroimidazol-1-yl)butyric acid* (**12a**). Following the general procedure. Yield: 82.0%. Faint yellow solid. m.p. 126.8–129.3 °C; ESI-MS *m*/*z* = 212.07 [M − H]^−^; ^1^H-NMR (600 MHz, DMSO-*d*_6_) δ 1.93–1.98 (m, 2H), 2.27 (t, *J* = 6.6 Hz, 2H), 2.36 (s, 3H), 4.01 (t, *J* = 6.6 Hz, 2H), 8.32 (s, 1H), 12.22 (br s, 1H).

*4-(2-Nitroimidazol-1-yl)butyric acid* (**12b**). Following the general procedure. Yield: 83.4%. Yellow solid. m.p. 111.3–113.2 °C; ESI-MS *m*/*z* = 198.06 [M − H]^−^; ^1^H-NMR (400 MHz, DMSO-*d*_6_) δ 2.00 (m, 2H), 2.26 (t, *J* = 7.2 Hz, 2H), 4.40 (t, *J* = 7.2 Hz, 2H), 7.16 (s, 1H), 7.65 (s, 1H), 12.14 (br s, 1H).

##### *General Synthetic Procedure of p-Toluenesulfonate Intermediates* (**13a**–**c**, **14a** *and* **15a**–**b**)

To a solution of **10a**–**c** (1.0 eq) in DMF, was added dropwise the solution of carbonyldiimidazole (CDI) (1.0 eq) in DMF. The mixture was stirred at room temperature for 1h. Then the solution of **5** (1.0 eq) and triethylamine (1.3 eq) in DMF was added dropwise into the first reaction mixture. The mixture was stirred under room temperature overnight. The reaction solution poured into water under vigorous mechanically stirring and filtered. The residue was dissolved in EA and the organic layer was separated, dried (anhydrous Na_2_SO_4_) and evaporated to dryness to afford the corresponding products (**14a**–**b**, **15a**–**b**) without further purification.

*N-[2-(2-Methyl-5-nitroimidazol-1-yl)acetyl]piperidin-4-yl-methyl p-toluenesulfonate* (**13a**). Following the general procedure. Yield: 89.5%. Brown jelly. ESI-MS *m*/*z* = 436.15 [M + H]^+^; ^1^H-NMR (400 MHz, DMSO-*d*_6_) δ 1.01 (m, 1H), 1.21 (m, 1H), 1.60 (d, *J* = 12.0 Hz, 1H), 1.68 (d, *J* = 12.8 Hz, 1H), 1.92 (m, 1H), 2.20 (s, 3H), 2.42 (s, 3H), 2.60 (t, *J* = 12.4 Hz, 1H), 3.04 (t, *J* = 12.4 Hz, 1H), 3.78 (d, *J* = 13.6 Hz, 1H), 3.91 (*J* = 6.4 Hz, 2H), 4.26 (d, *J* = 13.2 Hz, 1H), 5.04 (d, *J* = 17.2 Hz, 1H), 5.09 (d, *J* = 17.2 Hz, 1H), 7.48 (d, *J* = 8.4 Hz, 2H), 7.79 (d, *J* = 8.0 Hz, 2H), 8.14 (s, 1H).

*N-[2-(2-Nitroimidazol-1-yl)acetyl]piperidin-4-yl-methyl p-toluenesulfonate* (**13b**). Following the general procedure. Yield: 85.2%. Brown jelly. ESI-MS *m*/*z* = 423.14 [M + H]^+^; ^1^H-NMR (400 MHz, DMSO-*d*_6_) δ 0.96 (m, 1H), 1.20 (m, 1H), 1.58 (d, *J* = 12.4 Hz, 1H), 1.70 (d, *J* = 12.0 Hz, 1H), 1.93 (m, 1H), 2.42 (s, 3H), 2.60 (t, *J* = 12.0 Hz, 1H), 3.07 (t, *J* = 12.0 Hz, 1H), 3.80 (d, *J* = 13.6 Hz, 1H), 3.91 (*J* = 6.0 Hz, 2H), 4.22 (d, *J* = 12.8 Hz, 1H), 5.36 (d, *J* = 16.4 Hz, 1H), 5.40 (d, *J* = 16.4 Hz, 1H), 7.16 (s, 1H), 7.48 (d, *J* = 8.0 Hz, 2H), 7.53 (s, 1H), 7.79 (d, *J* = 8.0 Hz, 2H).

*N-[2-(5-Nitroimidazol-1-yl)acetyl]piperidin-4-yl-methyl p-toluenesulfonate* (**13c**). Following the general procedure. Yield: 91.2%. White powder. m.p. 112.5–115.1 °C; ESI-MS *m*/*z* = 423.14 [M + H]^+^; ^1^H-NMR (400 MHz, DMSO-*d*_6_) δ 1.00 (m, 1H), 1.20 (m, 1H), 1.59 (d, *J* = 12.4 Hz, 1H), 1.68 (d, *J* = 12.0 Hz, 1H), 1.91 (m, 1H), 2.42 (s, 3H), 2.59 (t, *J* = 12.0 Hz, 1H), 3.02 (t, *J* = 12.0 Hz, 1H), 3.77 (d, *J* = 13.6 Hz, 1H), 3.90 (*J* = 6.0 Hz, 2H), 4.27 (d, *J* = 13.2 Hz, 1H), 5.09 (d, *J* = 16.4 Hz, 1H), 5.13 (d, *J* = 16.8 Hz, 1H), 7.48 (d, *J* = 8.0 Hz, 2H), 7.71 (s, 1H), 7.78 (d, *J* = 8.0 Hz, 2H), 8.22 (s, 1H).

*N-[3-(2-Methyl-5-nitroimidazol-1-yl)propionyl]piperidin-4-yl-methyl p-toluenesulfonate* (**14a**). Following the general procedure. Yield: 82.2%. Brown jelly ESI-MS *m*/*z* = 451.18 [M + H]^+^; ^1^H-NMR (400 MHz, DMSO-*d*_6_) δ 0.90 (m, 1H), 1.04 (m, 1H), 1.57 (t, *J* = 12.8 Hz, 2H), 1.85 (m, 1H), 2.37 (s, 3H), 2.41 (s, 3H), 2.89 (t, *J* = 7.2 Hz, 2H), 3.79 (d, *J* = 13.6 Hz, 1H), 3.86 (d, *J* = 6.4 Hz, 2H), 4.13 (t, *J* = 7.2 Hz, 2H), 4.30 (t, *J* = 13.2 Hz, 1H), 7.47 (d, *J* = 8.8 Hz, 2H), 7.76 (d, *J* = 8.4 Hz, 2H), 8,27 (s,1H).

*N-[4-(2-Methyl-5-nitroimidazol-1-yl)butyryl]piperidin-4-yl-methyl p-toluenesulfonate* (**15a**). Following the general procedure. Yield: 78.6%. Faint yellow jelly. ESI-MS *m*/*z* = 465.18 [M + H]^+^; ^1^H-NMR (600 MHz, DMSO-*d*_6_) δ 0.89 (m, 1H), 1.14 (m, 1H), 1.58 (dd, *J*_1_ = 12.6 Hz, *J*_2_ = 30.0 Hz, 2H), 1.86 (m, 1H), 1.93 (t, *J* = 6.6 Hz, 2H), 2.32 (t, *J* = 6.6 Hz, 2H), 2.35 (s, 3H), 2.43 (s, 3H), 2.48 (t, *J* = 12.0 Hz, 1H), 2.91 (t, *J* = 12.0 Hz, 1H), 3.79 (d, *J* = 13.2 Hz, 1H), 3.91 (d, *J* = 5.4 Hz, 2H), 3.97 (t, *J* = 7.2 Hz, 2H), 4.33 (d, *J* = 12.6 Hz, 1H), 7.49(d, *J* = 7.8 Hz, 2H), 7.79 (d, *J* = 7.8 Hz, 2H), 8.31 (s, 1H).

*N-[4-(2-Nitroimidazol-1-yl)butyryl]piperidin-4-yl-methyl p-toluenesulfonate* (**15b**). Following the general procedure. Yield: 80.6%. Brownish jelly. ESI-MS *m*/*z* = 451.17 [M + H]^+^; ^1^H-NMR (600 MHz, DMSO-*d*_6_) δ 0.90 (m, 1H), 1.03 (m, 1H), 1.56 (m, 2H), 1.84 (m, 1H), 2.00 (m, 2H), 2.30 (t, *J* = 7.2 Hz, 2H), 2.41 (s, 3H), 2.43 (m, 1H), 2.89 (t, *J* = 12.0 Hz, 1H), 3.76 (d, *J* = 13.6 Hz, 1H), 3.87 (d, *J* = 6.0 Hz, 2H), 4.29 (d, *J* = 12.8 Hz, 1H), 4.38 (t, *J* = 7.2 Hz, 2H), 7.14 (s, 1H), 7.47(d, *J* = 8.0 Hz, 2H), 7.63 (s, 1H), 7.77 (d, *J* = 8.0 Hz, 2H).

##### *General Synthetic Procedure of Target Compounds* (**16a**–**p**)

To a solution of 4-anilino-6-methoxyquinazoline (**1a**–**g**) (1.0 eq) in DMF was added K_2_CO_3_. The mixture was stirred at 50 °C for 0.5 h. Then the solution of **13** (**14** or **15**, 1 eq) in DMF was added dropwise to the reaction mixture. The resulting mixture was stirred at 50 °C until no 4-anilino-6-methoxyquinazoline appeared (**1a**–**g**) on TLC. The reaction mixture was poured into water under vigorous stirring. Half an hour later, the mixture was filtrated. The residue was dissolved in methanol, and the organic layer was dried (anhydrous Na_2_SO_4_) and evaporated to dryness to afford a crude solid, which was washed by EA to afford the product.

*1-{4-[({4-[(4-Bromo-2-fluorophenyl)amino]-6-methoxyquinazolin-7-yl}oxy)methyl]piperidin-1-yl}-2-(2-methyl-5-nitroimidazol-1-yl)ethanone* (**16a**). Dark green powder prepared from **13a** and **1a** with the yield of 56.8%. m.p. 201.4–203.1 °C; ESI-MS *m*/*z* = 628.2, 630.2 [M + H]^+^, 626.3, 628.1 [M − H]^−^; ESI-HRMS calcd for C_27_H_28_BrFN_7_O_5_ *m*/*z* = 628.1319, 630.1299 [M + H]^+^, found: 628.1310, 630.1295; ^1^H-NMR (400 MHz, DMSO-*d*_6_) δ 1.24 (m, 1H), 1.42 (m, 1H), 1.89 (m, 2H), 2.17 (m, 1H), 2.24 (s, 3H), 2.74 (t, *J* = 12.0 Hz, 1H), 3.17 (t, *J* = 12.0 Hz, 1H), 3.87 (d, *J* = 12.8 Hz, 1H), 3.95 (s, 3H), 4.06 (d, *J* = 6.0 Hz, 2H), 4.36 (d, *J* = 12.4 Hz, 1H), 5.09 (d, *J* = 17.2 Hz, 1H), 5.16 (d, *J* = 17.2 Hz, 1H), 7.22 (s, 1H), 7.46 (d, 1H), 7.53 (t, 1H), 7.64 (d, 1H), 7.80 (s, 1H), 8.33 (s, 1H), 8.36 (s, 1H), 9.52(s, 1H); ^13^C-NMR (100 MHz, DMSO-*d*_6_) δ 13.15, 28.72, 29.41, 31.31, 35.64, 42.19, 44.64, 56.86, 72.98, 102.72, 108.49, 109.33, 119.86, 120.09, 124.10, 126.80, 128.13, 130.18, 145.69, 146.81, 147.60, 149.77, 153.60, 154.36, 157.56, 164.50; ^19^F-NMR (376 MHz, DMSO-*d*_6_) δ −115.47.

*1-{4-[({4-[(4-Bromo-2-fluorophenyl)amino]-6-methoxyquinazolin-7-yl}oxy)methyl]piperidin-1-yl}-3-(2-methyl-5-nitroimidazol-1-yl)propyl-1-one* (**16b**). Faint green powder prepared from **14a** and **1a** with the yield of 64.7%. m.p. 193.6–194.4 °C; ESI-MS *m*/*z* = 642.27, 644.25 [M + H]^+^; ESI-HRMS calcd for C_28_H_30_BrFN_7_O_5_ *m*/*z* = 642.1476, 644.1455 [M + H]^+^, found: 642.1473, 644.1457; ^1^H-NMR (600 MHz, DMSO-*d*_6_) δ 1.16 (m, 1H), 1.25 (m, 1H), 1.83 (m, 2H), 2.11 (m, 1H), 2.41 (s, 3H), 2.63 (t, *J* = 12.6 Hz, 1H), 2.95 (m, 2H), 3.04 (t, *J* = 12.6 Hz, 1H), 3.90 (d, *J* = 12.6 Hz, 1H), 3.95 (s, 3H), 4.01 (d, *J* = 6.0 Hz, 2H), 4.19 (t, *J* = 6.6 Hz, 2H), 4.43 (d, *J* = 12.6 Hz, 1H), 7.20 (s, 1H), 7.47 (d, 1H), 7.54 (t, 1H), 7.66 (d, 1H), 7.80 (s, 1H), 8.33 (s, 1H), 8.37 (s, 1H), 9.54 (s, 1H); ^13^C-NMR (151 MHz, DMSO-*d*_6_) δ 16.85, 28.13, 28.83, 32.59, 35.04, 40.71, 42.71, 44.36, 56.18, 72.31, 101.98, 107.74, 108.60, 117.46, 119.22, 122.15, 126.35, 127.46, 129.49, 145.26, 146.91, 149.04, 152.90, 155.78, 156.84, 157.44, 167.57; ^19^F-NMR (376 MHz, DMSO-*d*_6_) δ −115.46.

*1-{4-[({4-[(4-Bromo-2-fluorophenyl)amino]-6-methoxyquinazolin-7-yl}oxy)methyl]piperidin-1-yl}-4-(2-methyl-5-nitroimidazol-1-yl)butyl-1-one* (**16c**). Faint green powder prepared from **15a** and **1a** with the yield of 53.5%. m.p. 213.7–215.2 °C; ESI-MS *m*/*z* = 656.26, 658.22 [M + H]^+^; ESI-HRMS calcd for C_29_H_32_BrFN_7_O_5_ *m*/*z* = 656.1632, 658.1612 [M + H]^+^, found: 656.1629, 658.1612; ^1^H-NMR (600 MHz, DMSO-*d*_6_) δ 1.14 (m, 1H), 1.25 (m, 1H), 1.84 (t, *J* = 15.0 Hz, 2H), 1.96 (m, 2H), 2.10 (m, 1H), 2.39 (m, 5H), 2.60 (t, *J* = 12.0 Hz, 1H), 3.04 (t, *J* = 12.0 Hz, 1H), 3.89 (d, *J* = 12.6 Hz, 1H), 3.95 (s, 3H), 4.02 (m, 4H), 4.43 (d, *J* = 12.6 Hz, 1H), 7.20 (s, 1H), 7.47 (d, 1H), 7.54 (t, 1H), 7.66 (d, 1H), 7.81 (s, 1H), 8.34 (s, 1H), 8.37 (s, 1H), 9.54 (s, 1H); ^13^C-NMR (151 MHz, DMSO-*d*_6_) δ 12.49, 25.22, 28.14, 28.74, 35.15, 40.76, 44.40, 45.96, 56.10, 72.33, 101.97, 107.70, 108.61, 117.44, 119.20, 119.36, 122.00, 127.45, 129.48, 144.95, 145.38, 146.90, 149.05, 152.90, 153.65, 155.77, 156.85, 157.43, 169.15; ^19^F-NMR (376 MHz, DMSO-*d*_6_) δ −115.46.

*1-{4-[({4-[(4-Bromo-2-fluorophenyl)amino]-6-methoxyquinazolin-7-yl}oxy)methyl]piperidin-1-yl}-2-(2-nitroimidazol-1-yl)ethanone* (**16d**). Faint green powder prepared from **13b** and **1a** with the yield of 59.5%. m.p. 157.6–160.1 °C; ESI-MS *m*/*z* = 614.23, 616.20 [M + H]^+^; ESI-HRMS calcd for C_26_H_26_BrFN_7_O_5_ *m*/*z* = 614.1163, 616.1142 [M + H]^+^, found: 614.1157, 616.1142; ^1^H-NMR (400 MHz, DMSO-*d*_6_) δ 1.22 (m, 1H), 1.42 (m, 1H), 1.98 (dd, *J*_1_ = 12.0 Hz, *J*_2_ = 32.8 Hz, 2H), 2.17 (m, 1H), 2.74 (t, *J* = 11.6 Hz, 1H), 3.19 (t, *J* = 12.0 Hz, 1H), 3.88 (d, *J* = 12.0 Hz, 1H), 3.95 (s, 3H), 4.05 (d, *J* = 5.2 Hz, 2H), 4.32 (d, *J* = 12.0 Hz, 1H), 5.41 (d, *J* = 16.4 Hz, 1H), 5.47 (d, *J* = 16.4 Hz, 1H), 7.20 (d, 2H), 7.45 (d, 1H), 7.53 (m, 2H), 7.64 (d, 1H), 7.80 (s, 1H), 8.36 (s, 1H), 9.52 (s, 1H); ^13^C-NMR (100 MHz, DMSO-*d*_6_) δ 28.80, 29.48, 35.67, 42.24, 44.61, 51.73, 56.85, 72.96, 102.68, 108.49, 109.32, 118.15, 119.98, 127.10, 128.14, 129.10, 130.21, 145.92, 147.61, 149.78, 153.60, 154.37, 156.06, 157.56, 158.57, 164.34; ^19^F-NMR (376 MHz, DMSO-*d*_6_) δ −115.45.

*1-{4-[({4-[(4-Bromo-2-fluorophenyl)amino]-6-methoxyquinazolin-7-yl}oxy)methyl]piperidin-1-yl}-4-(2-nitroimidazol-1-yl)butyl-1-one* (**16e**). Faint green powder prepared from **15b** and **1a** with the yield of 52.5%. m.p. 192.1–193.8 °C; ESI-MS *m*/*z* = 642.22, 644.18 [M + H]^+^; ESI-HRMS calcd for C_28_H_30_BrFN_7_O_5_ *m*/*z* = 642.1476, 644.1455 [M + H]^+^, found: 642.1466, 644.1451; ^1^H-NMR (400 MHz, DMSO-*d*_6_):δ 1.15 (m, 1H), 1.26 (m, 1H), 1.81 (t, 2H), 2.05 (m, 3H), 2.38 (m, 2H), 2.57 (t, *J* = 11.6 Hz, 1H), 3.01 (t, *J* = 12.0 Hz, 1H), 3.85 (d, *J* = 13.2 Hz, 1H), 3.93 (s, 3H), 4.01 (d, *J* = 6.4 Hz, 2H), 4.41 (m, 3H), 7.18 (d, 2H), 7.43 (d, 1H), 7.50 (t, 1H), 7.64 (m, 2H), 7.80 (s, 1H), 8.34 (s, 1H), 9.56 (br, 1H); ^13^C-NMR (100 MHz, DMSO-*d*_6_) δ 25.94, 28.78, 29.57, 29.73, 35.87, 41.45, 45.15, 49.77, 56.84, 73.04, 102.75, 108.43, 109.37, 118.43, 109.37, 118.08, 119.84, 120.08, 128.14, 128.48, 130.16, 145.36, 147.55, 149.73, 153.56, 154.34, 156.04, 157.53, 158.54, 169.78; ^19^F-NMR (376 MHz, DMSO-*d*_6_) δ −115.42.

*1-{4-[({4-[(4-Bromo-2-fluorophenyl)amino]-6-methoxyquinazolin-7-yl}oxy)methyl]piperidin-1-yl}-2-(5-nitroimidazol-1-yl)ethanone* (**16f**). Dark green powder prepared from **13c** and **1a** with the yield of 64.5%. m.p. 144.6–146.1 °C; ESI-MS *m*/*z* = 614.24, 616.22 [M + H]^+^; ESI-HRMS calcd for C_26_H_26_BrFN_7_O_5_ *m*/*z* = 614.1163, 616.1142 [M + H]^+^, found: 614.1153, 616.1138; ^1^H-NMR (600 MHz, DMSO-*d*_6_) δ 1.27 (m, 1H), 1.41 (m, 1H), 1.90 (dd, *J*_1_ = 12.0 Hz, *J*_2_ = 31.8 Hz, 2H), 2.17 (m, 1H), 2.74 (t, *J* = 12.6 Hz, 1H), 3.16 (t, *J* = 12.6 Hz, 1H), 3.88 (d, *J* = 12.6 Hz, 1H), 3.97 (s, 3H), 4.06 (d, *J* = 6.6 Hz, 2H), 4.39 (d, *J* = 12.6 Hz, 1H), 5.17 (d, *J* = 16.2 Hz, 1H), 5.22 (d, *J* = 16.8 Hz, 1H), 7.23 (s, 1H), 7.47 (d, 1H), 7.55 (t, 1H), 7.64 (d, 1H), 7.77 (s, 1H), 7.82 (s, 1H), 8.29 (s, 1H), 8.38 (s, 1H), 9.56 (s, 1H); ^13^C-NMR (151 MHz, DMSO-*d*_6_) δ 27.97, 28.65, 34.93, 41.41, 43.81, 48.67, 56.16, 72.29, 102.01, 107.80, 108.64, 117.54, 119.37, 123.15, 127.47, 129.49, 138.50, 146.51, 146.91, 149.06, 152.92, 153.64, 155.78, 156.87, 157.45, 164.11; ^19^F-NMR (376 MHz, DMSO-*d*_6_) δ −115.46.

*1-{4-[({4-[(2-Chloro-4-fluorophenyl)amino]-6-methoxyquinazolin-7-yl}oxy)methyl]piperidin-1-yl}-2-(5-nitro-imidazol-1-yl)ethanone* (**16g**). Yellow solid powder prepared from **13c** and **1b** with the yield of 73.4%. m.p. 143.3–145.0 °C; ESI-HRMS calcd for C_26_H_26_ClFN_7_O_5_ *m*/*z* = 570.1668 [M + H]^+^, found: 570.1661; ^1^H-NMR (400 MHz, DMSO-*d*_6_) δ 1.24 (m, 1H), 1.41 (m, 1H), 1.89 (t, 2H), 2.16 (m, 1H), 2.73 (t, *J* = 12.0 Hz, 1H), 3.17 (t, *J* = 12.0 Hz, 1H), 3.88 (d, *J* = 12.8 Hz, 1H), 3.94 (s, 3H), 4.05 (d, *J* = 6.4 Hz, 2H), 4.37 (d, *J* = 12.8 Hz, 1H), 5.15 (d, *J* = 16.8 Hz, 1H), 5.19 (d, *J* = 16.4 Hz, 1H), 7.20 (s, 1H), 7.29 (td, *J*_1_ = 8.4 Hz, *J*_2_ = 2.8 Hz, 1H), 7.56 (m, 2H), 7.74 (s, 1H), 7.82 (s, 1H), 8.28 (d, *J* = 10.8 Hz, 2H), 9.51 (s, 1H); ^13^C-NMR (100 MHz, DMSO-*d*_6_) δ 27.97, 28.65, 34.92, 41.42, 43.82, 56.17, 72.30, 89.31, 102.04, 107.86, 108.43, 114.81, 116.95, 123.13, 127.55, 131.20, 132.20, 132.93, 138.49, 146.51, 146.78, 148.99, 153.05, 153.56, 157.61, 158.64, 161.09, 164.12; ^19^F-NMR (376 MHz, DMSO-*d*_6_) δ −114.05.

*1-{4-[({4-[(3-Bromo-4-methylphenyl)amino]-6-methoxyquinazolin-7-yl}oxy)methyl]piperidin-1-yl}-2-(5-nitro-imidazol-1-yl)ethanone* (**16h**). Green powder prepared from **13c** and **1c** with the yield of 66.6%. m.p. 150.6–152.3 °C; ESI-HRMS calcd for C_27_H_29_BrN_7_O_5_ *m*/*z* = 610.1414, 612.1393 [M + H]^+^, found: 610.1406, 612.1390; ^1^H-NMR (600 MHz, DMSO-*d*_6_) δ 1.24 (m, 1H), 1.39 (m, 1H), 1.92 (m, 2H), 2.15 (m, 1H), 2.33 (s, 1H), 2.73 (t, *J* = 12.0 Hz, 1H), 3.15 (t, *J* = 12.0 Hz, 1H), 3.86 (d, *J* = 12.8 Hz, 1H), 3.96 (s, 3H), 4.04 (d, *J* = 6.0 Hz, 2H), 4.36 (d, *J* = 12.8 Hz, 1H), 5.15 (d, *J* = 16.4 Hz, 1H), 5.19 (d, *J* = 16.4 Hz, 1H), 7.20 (s, 1H), 7.34 (d, *J* = 8.4 Hz, 1H), 7.75 (s, 1H), 7.79 (m, 2H), 8.13 (d, *J* = 1.6 Hz, 1H), 8.26 (s, 1H), 8.48 (s, 1H), 9.45 (s, 1H); ^13^C-NMR (151 MHz, DMSO-*d*_6_) δ 21.67, 27.97, 28.65, 34.94, 41.42, 43.82, 48.67, 56.31, 72.29, 101.93, 107.96, 108.81, 121.10, 123.12, 123.37, 124.84, 130.59, 131.49, 138.49, 138.76, 146.51, 146.91, 149.06, 152.70, 153.55, 156.03, 164.12.

*1-{4-[({4-[(2-Chloro-4-fluorophenyl)amino]-6-methoxyquinazolin-7-yl}oxy)methyl]piperidin-1-yl}-3-(2-methyl-5-nitro-imidazol-1-yl)propyl-1-one* (**16i**). Faint yellow powder prepared from **14a** and **1b** with the yield of 71.1%. m.p. 128.7–130.3 °C; ESI-HRMS calcd for C_28_H_30_ClFN_7_O_5_ *m*/*z* = 598.1981 [M + H]^+^, found: 598.1972; ^1^H-NMR (400 MHz, DMSO-*d*_6_) δ 1.18 (m, 2H), 1.83 (d, *J* = 11.6 Hz, 2H), 2.10 (m, 1H), 2.41 (s, 3H), 2.63 (t, *J* = 12.0 Hz, 1H), 2.95 (m, 2H), 3.04 (t, *J* = 12.0 Hz, 1H), 3.89 (m, 1H), 3.94 (s, 3H), 4.01 (d, *J* = 4.0 Hz, 2H), 4.19 (t, *J* = 6.8 Hz, 2H), 4.43 (d, *J* = 12.8 Hz, 1H), 7.19 (s, 1H), 7.31 (td, *J*_1_ = 8.4 Hz, *J*_2_ = 2.8 Hz, 1H), 7.51–7.67 (m, 2H), 7.83 (s, 1H), 8.32 (d, *J* = 11.6 Hz, 2H), 9.54 (br, 1H); ^13^C-NMR (100 MHz, DMSO-*d*_6_) δ 13.14, 28.62, 29.34, 33.09, 35.53, 41.21, 43.22, 44.86, 56.64, 72.80, 102.48, 108.28, 115.09, 117.46, 122.68, 132.65, 134.43, 145.78, 147.27, 149.45, 153.54, 154.04, 158.10, 168.08; ^19^F-NMR (376 MHz, DMSO-*d*_6_) δ −114.07.

*1-{4-[({4-[3,4-Difluorophenyl)amino]-6-methoxyquinazolin-7-yl}oxy)methyl]piperidin-1-yl}-3-(2-methyl-5-nitro-imidazol-1-yl)propyl-1-one* (**16j**). Faint yellow powder prepared from 14a and 1d with the yield of 63.9%. m.p. 174.6–177.1 °C; ESI-HRMS calcd for C_28_H_30_F_2_N_7_O_5_ *m*/*z* = 582.2776 [M + H]^+^, found: 582.2770; ^1^H-NMR (400 MHz, DMSO-*d*_6_) δ 1.19 (m, 2H), 1.82 (s, 2H), 2.08 (m, 1H), 2.41 (s, 3H), 2.61 (t, *J* = 12.0 Hz, 1H), 2.84–3.14 (m, 3H), 3.82–3.99 (m, 6H), 4.19 (t, *J* = 6.4 Hz, 2H), 4.43 (d, *J* = 12.0 Hz, 1H), 7.16 (s, 1H), 7.43 (dd, *J*_1_ = 19.2 Hz, *J*_2_ = 8.0 Hz, 1H), 7.58 (d, *J* = 8.0 Hz, 1H), 7.78 (s, 1H), 8.07 (dd, *J*_1_ = 11.6 Hz, *J*_2_ = 8.0 Hz, 1H), 8.32 (s, 1H), 8.49 (s, 1H), 9.53 (br, 1H); ^13^C-NMR (100 MHz, DMSO-*d*_6_) δ 13.08, 28.62, 29.32, 33.09, 35.57, 41.21, 43.19, 44.85, 56.73, 72.77, 102.27, 108.31, 109.20, 111.27, 111.48, 117.24, 117.42, 118.46, 122.62, 137.07, 137.16, 144.56, 145.73, 145.78, 146.83, 147.43, 147.94, 148.07, 149.52, 150.35, 150.48, 153.01, 154.05, 156.37, 168.05; ^19^F-NMR (376 MHz, DMSO-*d*_6_) δ −137.77 (d, *J_FF_* = 23.2 Hz), −145.10 (d, *J_FF_* = 23.2 Hz).

*1-{4-[({4-[(3-Bromo-4-methylphenyl)amino]-6-methoxyquinazolin-7-yl}oxy)methyl]piperidin-1-yl}-3-(2-methyl-5-nitro-imidazol-1-yl)propyl-1-one* (**16k**). Green powder prepared from **14a** and **1c** with the yield of 66.6%. m.p. 141.2–143.4 °C; ESI-HRMS calcd for C_29_H_33_BrN_7_O_5_ *m*/*z* = 638.1727, 640.1706 [M + H]^+^, found: 638.1716, 640.1701; ^1^H-NMR (400 MHz, DMSO-*d*_6_) δ 1.17 (m, 2H), 1.81 (s, 2H), 2.08 (m, 1H), 2.32 (s, 3H), 2.41 (s, 3H), 2.60 (t, *J* = 12.0 Hz, 1H), 2.88–3.04 (m, 3H), 3.88–3.96 (m, 6H), 4.18 (s, 2H), 4.43 (d, *J* = 12.0 Hz, 1H), 7.14 (s, 1H), 7.32 (d, *J* = 8.0 Hz, 1H), 7.81 (d, 2H), 8.16 (s, 1H), 8.32 (s, 1H), 8.49 (s, 1H), 9.45 (br, 1H); ^13^C-NMR (100 MHz, DMSO-*d*_6_) δ 13.11, 22.16, 28.62, 29.33, 33.10, 35.57, 43.19, 44.86, 56.74, 72.74, 102.32, 108.28, 109.27, 121.49, 122.64, 123.86, 125.24, 131.00, 131.85, 139.51, 145.73, 145.78, 147.37, 149.46, 153.32, 153.95, 156.44, 168.03.

*1-{4-[({4-[3-Fluorophenyl)amino]-6-methoxyquinazolin-7-yl}oxy)methyl]piperidin-1-yl}-3-(2-methyl-5-nitro-imidazol-1-yl)propyl-1-one* (**16l**). Faint yellow powder prepared from **14a** and **1e** with the yield of 63.3%. m.p. 175.8–178.3 °C; ESI-HRMS calcd for C_28_H_31_FN_7_O_5_ *m*/*z* = 564.2371 [M + H]^+^, found: 564.2375; ^1^H-NMR (400 MHz, DMSO-*d*_6_) δ 1.18 (m, 2H), 1.82 (s, 2H), 2.08 (m, 1H), 2.41 (s, 3H), 2.61 (t, *J* = 12.0 Hz, 1H), 2.96–3.02 (m, 3H), 3.89–3.97 (m, 6H), 4.19 (s, 2H), 4.43 (d, *J* = 12.0 Hz, 1H), 6.91 (s, 1H), 7.17 (s, 1H), 7.41 (d, *J* = 4.0 Hz, 1H), 7.63 (d, *J* = 8.0 Hz, 1H), 7.82 (s, 1H), 7.92 (d, *J* = 11.6 Hz, 1H), 8.32 (s, 1H), 8.52 (s, 1H), 9.56 (s, 1H); ^13^C-NMR (100 MHz, DMSO-*d*_6_) δ 13.10, 28.62, 29.32, 33.09, 35.56, 41.21, 43.19, 44.85, 56.77, 72.77, 102.36, 108.33, 108.83, 109.09, 109.38, 109.70, 109.91, 117.82, 122.63, 130.22, 141.91, 142.02, 145.74, 145.78, 147.51, 149.54, 153.05, 154.06, 156.44, 161.31, 163.70, 168.05; ^19^F-NMR (376 MHz, DMSO-*d*_6_) δ −112.66.

*1-{4-[({4-[4-Fluorophenyl)amino]-6-methoxyquinazolin-7-yl}oxy)methyl]piperidin-1-yl}-3-(2-methyl-5-nitro-imidazol-1-yl)propyl-1-one* (**16m**). Faint yellow powder prepared from **14a** and **1f** with the yield of 59.1%. m.p. 171.3.3–172.7 °C; ESI-HRMS calcd for C_28_H_31_FN_7_O_5_ *m*/*z* = 564.2371 [M + H]^+^, found: 564.2373; ^1^H-NMR (400 MHz, DMSO-*d*_6_) δ 1.17 (m, 2H), 1.82 (d, *J* = 11.6 Hz, 2H), 2.08 (m, 1H), 2.41 (s, 3H), 2.61 (t, *J* = 12.0 Hz, 1H), 2.90–3.06 (m, 3H), 3.90 (d, *J* = 12.4 Hz, 1H), 3.97 (m, 5H), 4.19 (t, *J* = 6.8 Hz, 2H), 4.43 (d, *J* = 12.4 Hz, 1H), 7.17 (s, 1H), 7.23 (t, *J* = 8.0 Hz, 2H), 7.80 (m, 3H), 8.33 (s, 1H), 8.44 (s, 1H), 9.50 (s, 1H); ^13^C-NMR (100 MHz, DMSO-*d*_6_) δ 13.11, 28.62, 29.33, 33.09, 35.57, 41.21, 43.20, 44.85, 56.73, 72.15, 102.44, 108.33, 109.17, 115.33, 115.55, 122.65, 124.76, 124.81, 136.19, 136.22, 145.75, 145.77, 147.33, 149.41, 153.30, 153.92, 156.80, 157.56, 159.95, 168.05; ^19^F-NMR (376 MHz, DMSO-*d*_6_) δ −119.21.

*1-{4-[({4-[(3,4-Dichloro-2-fluorophenyl)amino]-6-methoxyquinazolin-7-yl}oxy)methyl]piperidin-1-yl}-3-(2-methyl-5-nitro-imidazol-1-yl)propyl-1-one* (**16n**). Faint yellow powder prepared from **14a** and **1g** with the yield of 68.6%. m.p. 157.1–159.5 °C; ESI-HRMS calcd for C_28_H_29_Cl_2_FN_7_O_5_ *m*/*z* = 632.1591 [M + H]^+^, found: 632.1586; ^1^H-NMR (400 MHz, DMSO-*d*_6_) δ 1.19 (m, 2H), 1.82 (s, 2H), 2.08 (m, 1H), 2.41 (s, 3H), 2.61 (t, *J* = 12.0 Hz, 1H), 2.84–3.14 (m, 3H), 3.82–3.99 (m, 6H), 4.19 (t, *J* = 6.4 Hz, 2H), 4.43 (d, *J* = 12.0 Hz, 1H), 7.19 (m, 1H), 7.57 (m, 2H), 7.81 (s, 1H), 8.32 (s, 1H), 8.44 (s, 1H), 9.78 (s, 1H); ^13^C-NMR (100 MHz, DMSO-*d*_6_) δ 13.15, 22.17, 28.65, 29.33, 33.11, 35.56, 43.20, 44.86, 56.71, 72.72, 102.40, 108.19, 109.15, 119.98, 120.19, 125.78, 127.24, 127.92, 128.92, 145.72, 149.67, 152.28, 153.27, 154.31, 154.76, 157.17, 164.18; ^19^F-NMR (376 MHz, DMSO-*d*_6_) δ −113.50.

*1-{4-[({4-[(2-Chloro-4-fluorophenyl)amino]-6-methoxyquinazolin-7-yl}oxy)methyl]piperidin-1-yl}-2-(2-nitro-imidazol-1-yl)ethanone* (**16o**). Faint yellow powder prepared from **13b** and **1b** with the yield of 75.7%. m.p. 158.6–159.9 °C; ESI-HRMS calcd for C_26_H_36_ClFN_7_O_5_ *m*/*z* = 570.1668 [M + H]^+^, found: 570.1661; ^1^H-NMR (400 MHz, DMSO-*d*_6_) δ 1.19 (m, 1H), 1.41 (m, 1H), 1.83 (d, *J* = 11.6 Hz, 1H), 1.92 (d, *J* = 11.6 Hz, 1H), 2.16 (m, 1H), 2.73 (t, *J* = 12.0 Hz, 1H), 3.18 (t, *J* = 12.0 Hz, 1H), 3.91 (d, *J* = 12.0 Hz, 1H), 3.97 (s, 3H), 4.03 (d, *J* = 5.6 Hz, 2H), 4.31 (d, *J* = 12.0 Hz, 1H), 5.46 (d, *J* = 16.0 Hz, 1H), 5.53 (d, *J* = 16.0 Hz, 1H), 7.20 (d, 2H), 7.29 (td, *J*_1_ = 9.6 Hz, *J*_2_ = 2.8 Hz 1H), 7.54 (m, 2H), 7.66 (s, 1H), 8.09 (s, 1H), 8.33 (s, 1H), 10.15 (br, 1H); ^13^C-NMR (100 MHz, DMSO-*d*_6_) δ 28.61, 29.28, 35.43, 42.04, 44.45, 51.57, 57.01, 72.81, 103.42, 107.36, 108.88, 115.03, 115.25, 117.16, 117.42, 125.91, 127.87, 128.68, 128.97, 131.62, 132.77, 133.43, 145.70, 149.53, 152.97, 154.21, 158.30, 159.16, 161.61, 164.18; ^19^F-NMR (376 MHz, DMSO-*d*_6_) δ −113.92.

*1-{4-[({4-[(3,4-Dichloro-2-fluorophenyl)amino]-6-methoxyquinazolin-7-yl}oxy)methyl]piperidin-1-yl}-2-(2-nitro-imidazol-1-yl)ethanone* (**16p**). Faint yellow powder prepared from **13b** and **1g** with the yield of 70.3%. m.p. 166.6–168.7 °C; ESI-HRMS calcd for C_26_H_25_Cl_2_FN_7_O_5_ *m*/*z* = 604.1278 [M + H]^+^, found: 604.1272; ^1^H-NMR (400 MHz, DMSO-*d*_6_) δ 1.24 (m, 1H), 1.43 (m, 1H), 1.86 (d, *J* = 11.6 Hz, 1H), 1.94 (d, *J* = 11.6 Hz, 1H), 2.18 (m, 1H), 2.75 (t, *J* = 12.0 Hz, 1H), 3.21 (t, *J* = 12.0 Hz, 1H), 3.92 (d, *J* = 12.0 Hz, 1H), 3.97 (s, 3H), 4.07 (d, *J* = 5.6 Hz, 2H), 4.35 (d, *J* = 12.0 Hz, 1H), 5.43 (d, *J* = 16.0 Hz, 1H), 5.49 (d, *J* = 16.0 Hz, 1H), 7.21 (m, 2H), 7.59 (m, 3H), 7.81 (s, 1H), 8.42 (s, 1H), 9.73 (s, 1H); ^13^C-NMR (100 MHz, DMSO-*d*_6_) δ 28.63, 29.29, 35.49, 42.05, 44.43, 51.55, 56.65, 72.79, 102.42, 108.21, 109.14, 119.99, 120.18, 125.78, 127.23, 127.91, 128.92, 145.73, 149.67, 152.28, 153.27, 154.29, 154.77, 157.13, 164.15; ^19^F-NMR (376 MHz, DMSO-*d*_6_) δ −113.50.

### 3.2. Molecular Docking Study

Two docking studies of compound **16f** with EGFR (PDB ID: 4I23) and VEFR-2 (PDB ID: 2RL5) were performed using vandetanib as a comparison. The molecular docking procedure was performed by the Glide Dock method using Schrödinger software (Schrödinger Corp., New York, NY, USA). For ligand preparation, the 3D structure of compound **16f** was generated and minimized. For enzyme preparation, the hydrogen atoms were added and the Chemistry at HARvard Macromolecular Mechanics (CHARMM) force field was employed. EGFR and VEFR-2 were defined as receptors and the site spheres were selected based on the ligand binding location of vandetanib, then vandetanib was removed and compound **16f** was placed by the MacroModel module through Embrace Minimization procedure. Types of interactions of the docked receptors with ligands were analyzed after end of molecular docking. Ten docking poses were saved for each ligand and the final docked conformations were evaluated by the score of the program and also by visual inspection.

### 3.3. Pharmacology

All cell lines mentioned above were purchased from Cell Bank of China Science Academy (Beijing, China). The above cells were cultured in RPMI-1640 (Gibco Corp., USA) with 10% fetal calf serum. MTS assay kit (Promega Corp., Madison, WI, USA) and CCK-8 assay kit (BestBio Corp., Shanghai, China) were used in corresponding assays. Primer sequences of VEGF, EGF, and β-actin (Sangon Corp., Shanghai, China) were used for VEGF/EGF gene expression inhibition tests. Cobalt chloride hexahydrate was purchased from Sigma-Aldrich (St. Louis, MO, USA). All of the test compounds were dissolved in DMSO for use.

#### 3.3.1. In Vitro EGFR Inhibitory Assay

The In Vitro EGFR Inhibitory activities of compound **16a**–**f** on A431 cells and H1975 cells were determined by MTS method. The cells were incubated with 10% RPMI-1640 medium at 37 °C in 5% CO_2_ and seeded in 96-well tissue culture plates at the concentration of 1 × 10^4^ cells per well. The cells adhered completely after 24 h of incubation. Removed the medium and washed with d-hanks buffer, then exposed cells to different concentrations of test compounds (0.3, 1, 3, 10, 30, and 100 μmol/L in DMSO) dissolved in non-serum medium (three wells per concentration with corresponding blank groups). 0.1% DMSO was added to the control group. After 48 h of incubation at 37 °C in 5% CO_2_, added 20 μL MTS solution to per well and incubated for another 0.5 h. Absorbance was measured at 490 nm in plate reader (RT-6100 Rayto, Shenzhen, China) and calculated for inhibition ratios and IC_50_ values.

#### 3.3.2. In Vitro Anti-Proliferative Activity Assays in Normoxia and Hypoxia

A549 and H446 cells were divided into hypoxic and normoxic groups (Hela cells were only determined in hypoxia). Both groups were incubated and exposed to two concentrations of test compounds (0.5 and 5 μmol/L in DMSO) in the same way with above assay. In hypoxic groups, cobalt (II) chloride solution (100 μmol/L in water) was added, except for blank groups. After 48 h of incubation at 37 °C in 5% CO_2_, 20 μL CCK-8 solution was added to per well and incubated for another 1 h. Measured absorbance at 450 nm in plate reader and calculated for inhibition ratios.

#### 3.3.3. In Vitro VEGF/EGF Gene Expression Inhibition Assays

Two groups of A549 cells were seeded in 96-well tissue culture plates at the concentration of 1 × 10^5^ cells per well. Cobalt (II) chloride solution (100 μmol/L) (containing 10% serum HG-DMEM) was added to cells and setup blank and control groups. Cells were exposed to two concentrations of test compounds (0.5 and 5 μmol/L in DMSO, three wells per concentration) and extracted RNAs by the Trizol method after 24 h of incubation. The Q-PCR method was used for determining the amounts of VEGF mRNA and EGF mRNA, with β-actin as internal control.

## 4. Conclusions

In summary, sixteen novel compounds were designed and synthesized by incorporating the nitroimidazole moieties into the piperidine N-4 position of vandetanib with amide linkers. The molecular docking study of **16f** with EGFR and VEFR-2 has revealed that it could fit well into EGFR and VEFR-2 kinase in a similar way to vandetanib and an extra H-bond formed between the nitroimidazole group and Lys838 residue at the opening of the hydrophobic pocket in VEGFR-2, notwithstanding that it did not seem very firm, with a distance of 3.491 Å. More than a half of the compounds showed acceptable EGFR inhibition activities over wild-type tumor cells but obviously weak activities over T790M mutant tumor cells. In in vitro normoxic/hypoxic anti-proliferative assays, all compounds demonstrated superior activities to vandetanib in hypoxia and the activities were greatly activated at low concentration comparing with normoxia. In VEGF/EGF gene expression inhibition assays, all target compounds exhibited excellent VEGF expression inhibitory activities in hypoxia and were distinctly superior to vandetanib; nevertheless, EGF gene expression levels are augmented mostly in hypoxia. Among target compounds, **16f** showed the most potent activities in the above-mentioned assays and was worthy of further development as a potential cancer therapeutic agent in hypoxia.

## Figures and Tables

**Table 1:** In vitro EGFR inhibitory activity assay results of target compounds **16a**–**p**.

Compound	Substituent						IC_50_ (μmol/L)	
	R_1_	R_2_	n	X_1_	X_2_	X_3_	A431	H1975
**16a**	CH_3_	NO_2_	1	F	H	Br	1.82	58.46
**16b**	CH_3_	NO_2_	2	F	H	Br	3.42	49.52
**16c**	CH_3_	NO_2_	3	F	H	Br	2.03	53.42
**16d**	NO_2_	H	1	F	H	Br	2.10	60.12
**16e**	NO_2_	H	3	F	H	Br	4.81	40.15
**16f**	H	NO_2_	1	F	H	Br	1.64	60.01
**16g**	H	NO_2_	1	Cl	H	F	39.76	71.80
**16h**	H	NO_2_	1	H	Br	CH_3_	12.34	78.25
**16i**	CH_3_	NO_2_	2	Cl	H	F	15.14	78.82
**16j**	CH_3_	NO_2_	2	H	F	F	65.42	87.90
**16k**	CH_3_	NO_2_	2	H	Br	CH_3_	17.95	74.84
**16l**	CH_3_	NO_2_	2	H	F	H	78.94	>100
**16m**	CH_3_	NO_2_	2	H	H	F	66.75	>100
**16n**	CH_3_	NO_2_	2	F	Cl	Cl	6.75	46.22
**16o**	NO_2_	H	1	Cl	H	F	24.84	58.55
**16p**	NO_2_	H	1	F	Cl	Cl	9.45	37.85
**Vadetanib**	-	-	-	F	H	Br	0.85	4.81

**Table 2:** In vitro anti-proliferative activity assay results of target compounds in normoxia and hypoxia ^a^.

			Compound										
			Vandet-anib	16a	16b	16c	16d	16e	16f	16h	16i	16n	16p
5 μM inhibition ratio (%)	A549	Normoxia	37.30	40.43	37.32	43.78	46.74	66.09	12.02	33.17	38.76	42.23	55.84
		Hypoxia	40.82	46.64	57.95	71.04	41.33	85.70	52.95	65.81	50.29	56.11	80.33
	H446	Normoxia	27.09	47.07	41.56	35.36	45.57	51.87	30.00	39.20	36.92	31.53	49.60
		Hypoxia	43.01	44.81	58.84	48.42	60.77	68.21	45.74	56.60	53.64	57.32	70.19
	Hela ^b^	Hypoxia	36.43	10.83	26.21	21.40	18.19	23.46	8.00	13.66	18.93	10.53	36.24
0.5 μM inhibition ratio (%)	A549	Normoxia	11.22	4.02	6.81	15.24	11.20	24.43	0.52	15.42	7.04	12.75	22.67
		Hypoxia	29.95	58.08	69.30	69.93	17.62	50.97	62.01	25.70	30.87	47.96	47.13
	H446	Normoxia	12.53	16.95	8.97	11.44	17.92	22.31	3.69	28.79	12.79	15.21	26.04
		Hypoxia	37.41	42.33	62.94	62.52	53.82	43.74	59.86	36.06	47.72	52.92	42.98
	Hela ^b^	Hypoxia	14.78	8.09	15.91	21.01	15.48	18.47	11.59	<0	10.54	12.44	18.55

^a^ Control group in hypoxia was performed with 200 μmol/L cobalt chloride solution and corresponding amount of DMSO. ^b^ Anti-proliferative activity test over HeLa cells in normoxia was not determined.

**Table 3:** VEGF/EGF gene expression level assay results of target compounds **16a**–**f**.

		Compound							
		Control ^b,c^	Vandetanib	16a	16b	16c	16d	16e	16f
VEGF gene expression level	Normoxia	1.0000	0.6457	0.1070	0.1387	0.0949	0.0606	0.1866	0.0706
	Hypoxia **^a^**	1.0000	0.2457	0.0009	0.0009	0.0007	0.0008	0.0006	0.0002
EGF gene expression level	Normoxia	1.0000	0.3440	0.5260	0.9732	0.5471	0.4366	1.1758	0.5614
	Hypoxia **^a^**	1.0000	5.4907	2.5398	2.8108	1.7447	1.9433	2.4923	0.3199

**^a^** Concentration of cobalt chloride in control and experimental groups was 200 μmol/L. **^b^** Control group was performed with a corresponding amount of DMSO. **^c^** VEGF/EGF gene expression levels in control groups in hypoxia or normoxia was calculated as one, respectively and independently.
